# Supplementary material for: Environmental heterogeneity modulates the effect of plant diversity on the spatial variability of grassland biomass
Source: Nat Commun. 2023 Mar 31;14:1809. doi: 10.1038/s41467-023-37395-y (PMC10066197; doi:10.1038/s41467-023-37395-y)
Supplement: Supplementary file 1 — Supplementary Information [file 41467_2023_37395_MOESM1_ESM.pdf]

**SUPPLEMENTARY INFORMATION**

# Environmental heterogeneity modulates the effect of plant diversity on the spatial variability of grassland biomass

Pedro Daleo[1], Juan Alberti[1], Enrique J. Chaneton[†], Oscar Iribarne[1], Pedro M. Tognetti[2], Jonathan D. Bakker[3], Elizabeth T. Borer[4], Martín Bruschetti[1], Andrew S. MacDougall[5], Jesús Pascual[1], Mahesh Sankaran[6,7], Eric W. Seabloom[4], Shaopeng Wang[8], Sumanta Bagchi[9], Lars A. Brudvig[10], Jane A. Catford[11,12], Chris R. Dickman[13], Timothy L. Dickson[14], Ian Donohue[15], Nico Eisenhauer[16,17], Daniel S. Gruner[18], Sylvia Haider[19,16,20], Anke Jentsch[21], Johannes M. H. Knops[22], Ylva Lekberg[23], Rebecca L. McCulley[24], Joslin L. Moore[25,26,12], Brent Mortensen[27], Timothy Ohlert[28], Meelis Pärtel[29], Pablo L. Peri[30], Sally A. Power[31], Anita C. Risch[32], Camila Rocca[1], Nicholas G. Smith[33], Carly Stevens[34], Riin Tamme[29], G.F. (Ciska) Veen[35], Peter A.Wilfahrt[4], Yann Hautier[36]

[1] Instituto de Investigaciones Marinas y Costeras (IIMyC), UNMDP – CONICET, CC 1260 Correo Central, B7600WAG, Mar del Plata, Argentina; [2] IFEVA-Facultad de Agronomía, Universidad de Buenos Aires - CONICET, Av San Martín 4453 C1417DSE, Ciudad Autónoma de Buenos Aires, Argentina; [3] School of Environmental and Forest Sciences, University of Washington, Seattle, WA 98195, USA; [4] Department of Ecology, Evolution & Behavior, University of Minnesota, St. Paul, MN 55108, USA; [5] Department of Integrative Biology, University of Guelph, Guelph, Ontario, Canada N1G2W1; [6] National Centre for Biological Sciences, Tata Institute of Fundamental Research, Bengaluru 560065, Karnataka, India; [7] School of Biology, University of Leeds, Leeds LS2 9JT, UK; [8] Institute of Ecology, College of Urban and Environmental Science, and Key Laboratory for Earth Surface Processes of the Ministry of Education, Peking University, 100871, Beijing, China; [9] Centre for Ecological Sciences, Indian Institute of Science, Bangalore, 560012, India; [10] Department of Plant Biology and Program in Ecology, Evolution, and Behavior, Michigan State University, East Lansing, MI 48824 USA; [11] Department of Geography, King's College London, 30 Aldwych, London, WC2B 4BG, UK; [12] School of Ecosystem and Forest Sciences, University of Melbourne, Parkville, Victoria 3010, Australia; [13] Desert Ecology Research Group, School of Life & Environmental Sciences, University of Sydney, NSW 2006, Australia; [14] University of Nebraska at Omaha, Department of Biology, Omaha, NE, USA; [15] Zoology, School of Natural Sciences, Trinity College Dublin, Dublin 2, Ireland; [16] German Centre for Integrative Biodiversity Research (iDiv) Halle-Jena-Leipzig, Leipzig, Germany; [17] Institute of Biology, Leipzig University, Germany; [18] Department of Entomology, University of Maryland, College Park, MD 20742 USA; [19] Institute of Biology / Geobotany and Botanical Garden, Martin Luther University Halle-Wittenberg, Halle, Germany; [20] Institute of Ecology, Leuphana University of Lüneburg, Lüneburg, Germany; [21] Disturbance Ecology, BayCEER, University of Bayreuth, 95447 Bayreuth, Germany; [22] Department of Health & Environmental Sciences, Xi'an Jiaotong-Liverpool University, Suzhou, Jiangsu, China; [23] MPG Ranch and University of Montana, W.A. Franke College of Forestry and Conservation, Missoula, MT, 59812, USA; [24] Department of Plant and Soil Sciences, University of Kentucky, Lexington, KY, 40546, USA; [25] Arthur Rylah Institute for Environmental Research, 123 Brown Street, Heidelberg, Victoria 3084, Australia; [26] School of Biological Sciences, Monash University, 25 Rainforest Walk, Clayton, Victoria 3800, Australia; [27] Department of Biology, Benedictine College, KS, USA; [28] Department of Biology, Colorado State University, CO, USA; [29] Institute of Ecology and Earth Sciences, University of Tartu, Estonia; [30] Instituto Nacional de Tecnología Agropecuaria (INTA)- Universidad Nacional de la Patagonia Austral (UNPA) -

CONICET. Río Gallegos, Santa Cruz, Argentina; [31] Hawkesbury Institute for the Environment, Western Sydney University, Locked Bag 1797, Penrith, New South Wales 2751, Australia; [32] Swiss Federal Institute for Forest, Snow and Landscape Research WSL, Community Ecology, Zuercherstrasse 111, 8903 Birmensdorf, Switzerland; [33] Texas Tech University, 2901 Main St., Lubbock, TX, USA 79409; [34] Lancaster Environment Centre, Lancaster University, Lancaster, LA1 4YQ, UK; [35] Department of Terrestrial Ecology, Netherlands Institute of Ecology, PO Box 50, 6700 AB Wageningen, The Netherlands; [36] Ecology and Biodiversity Group, Department of Biology, Utrecht University, Padualaan 8, 3584 CH Utrecht, The Netherlands; [†] Deceased on March 2019

# Supplementary Fig. 1

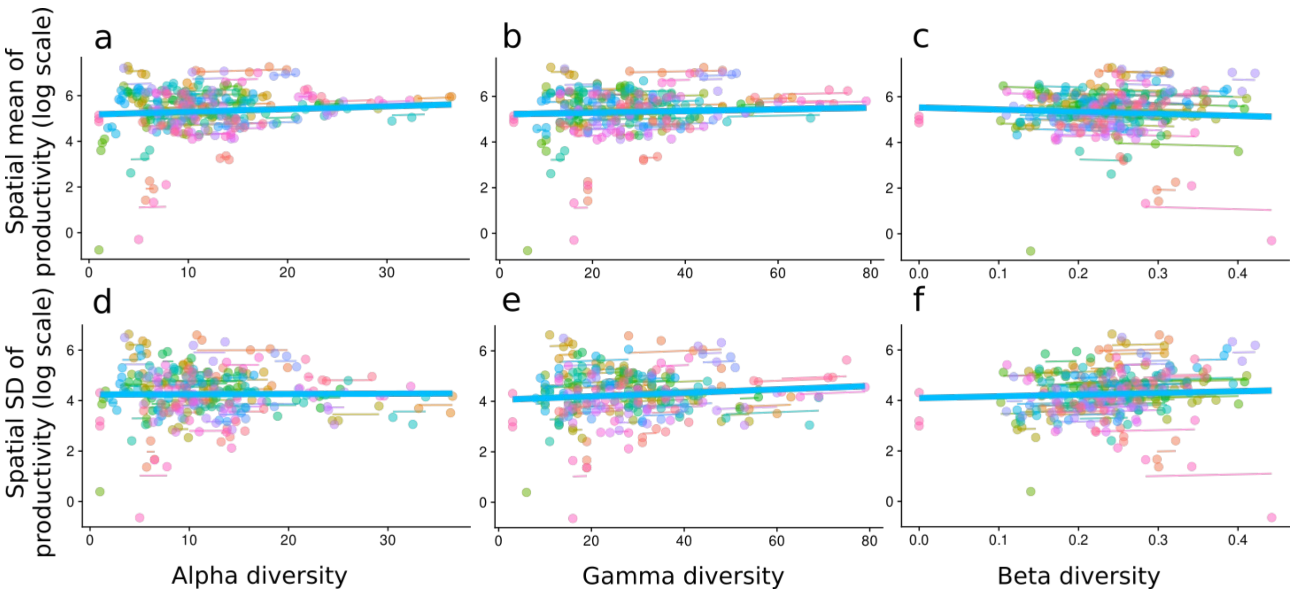


**Supplementary Fig. 1. The relationships between plant species diversity and the two separate components of spatial variability (i.e. μ, the mean plot biomass and σ, the standard deviation of plot biomass.** There was no significant relation between any scale of diversity and the mean plot biomass: **a** Alpha diversity (slope and 95% CIs= 0.006 (-0.011 to 0.024)); **b** gamma diversity (slope and 95% CIs= 0.002 (-0.005 to 0.009); and **c** beta diversity (slope and 95% CIs= -0.398 (-1.305 to 0.508). There was also no relation between alpha and gamma diversity and the SD of plot biomass: **d** Alpha diversity (slope and 95% CIs= -0.002 (-0.024 to 0.020)); **e** gamma diversity (slope and 95% CIs= 0.006 (-0.004 to 0.015); **f** beta diversity, nevertheless, was positively associated with the SD of plot biomass (slope and 95% CIs= 1.570 (0.131 to 3.001). Different colors represent different sites (See Fig 2 for site color key assignment), major lines (in turquoise) represent the fixed-effect linear regression slopes among sites and small colored lines show patterns within sites. Inference was based on 95% confidence intervals of the slopes and on the Likelihood Ratio Test, that tests the difference in two nested models (in this case with and without the diversity index to be evaluated) using the Chi square distribution (two-sided test).

**Supplementary Fig. 2**

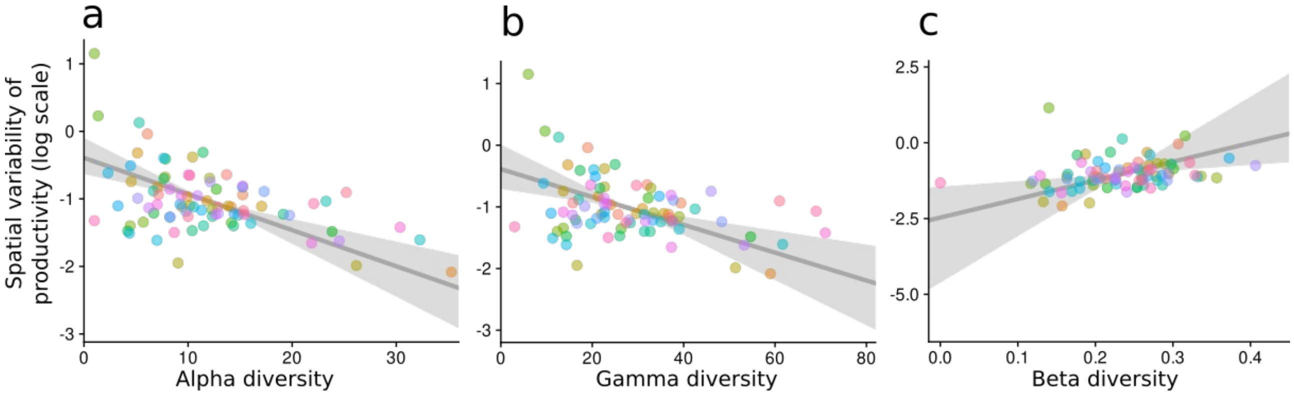


**Supplementary Fig. 2. The relation between plant species diversity and spatial variability of biomass production among 83 globally distributed grasslands sites of the Nutrient Network analyzed with type II regression. a** The relationship between mean plot species diversity (alpha diversity) and spatial variability of productivity was negative ($r^2$= 0.23, slope (95% confidence intervals)= -0.053 (-0.078 to -0.033), P= 0.001); **b** Total block species diversity (gamma diversity) was also negatively associated with spatial variability of productivity ($r^2$= 0.14, slope (95% confidence intervals)= -0.023 (-0.037 to -0.011), P= 0.001); **c** Plot to plot variation in species composition (beta diversity), in contrast, was positively associated with spatial variability of biomass production ($r^2$= 0.06, slope (95% confidence intervals)= 6.14 (1.81 to 15.22), P= 0.01). For **a-c**, gray lines represent the linear regression slopes and the shaded zone around the line represents 95% confidence interval. See Fig. 2 for site color key assignment. Inference was based on 95% confidence intervals of the slopes and on the permutational probability (one-tailed, for the tail corresponding to the sign of the slope estimate).

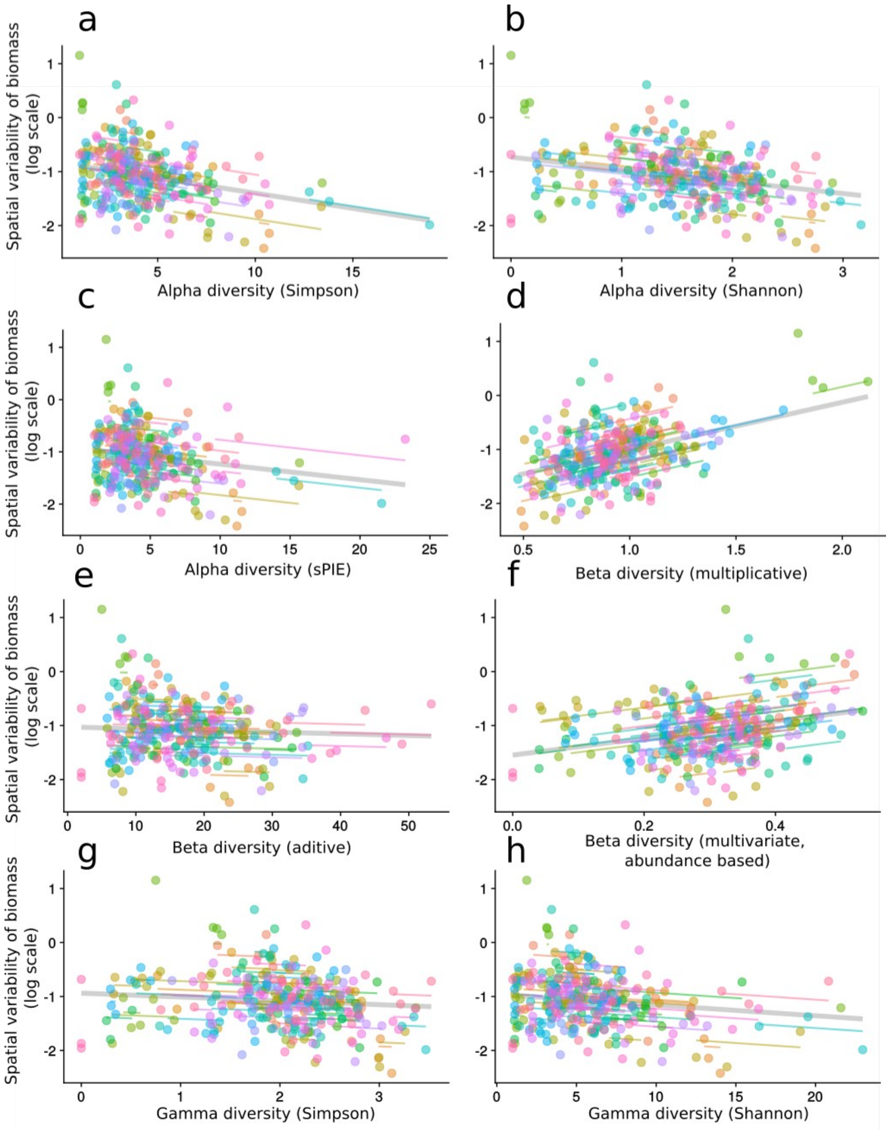


**Supplementary Fig. 3. Relationships between different indexes of diversity and spatial variability of biomass production.** The spatial variability of biomass production (measured as standard deviation/mean of biomass production; natural log transformed) of 83 grassland sites of the Nutrient Network was negatively associated with alpha species diversity measured as: **a** Inverse Simpson diversity index; **b** Shannon diversity index and **c** Effective number of species needed to reach the observed Probability of Interspecific Encounter (Spie). The spatial variability of biomass production was positively associated with beta diversity measured as multiplicative (i.e. gamma/alpha) beta diversity **d** and abundance-based multivariate beta diversity **f** but not as additive (i.e. gamma-alpha) beta diversity **e.** It was also negatively associated with gamma diversity measured as **g** Inverse Simpson diversity index but not as **h** Shannon diversity index (see Supplementary Table S1 for effect sizes and 95% CIs). Different colors represent different sites (See Fig. 2 for site color key assignment), major lines (in turquoise) represent the fixed-effect linear regression slopes among sites and small colored lines show patterns within sites. Inference was based on 95% confidence intervals of the slopes and on the Likelihood Ratio Test, that tests the difference in two nested models (in this case with and without the diversity index to be evaluated) using the Chi square distribution (two-sided test).

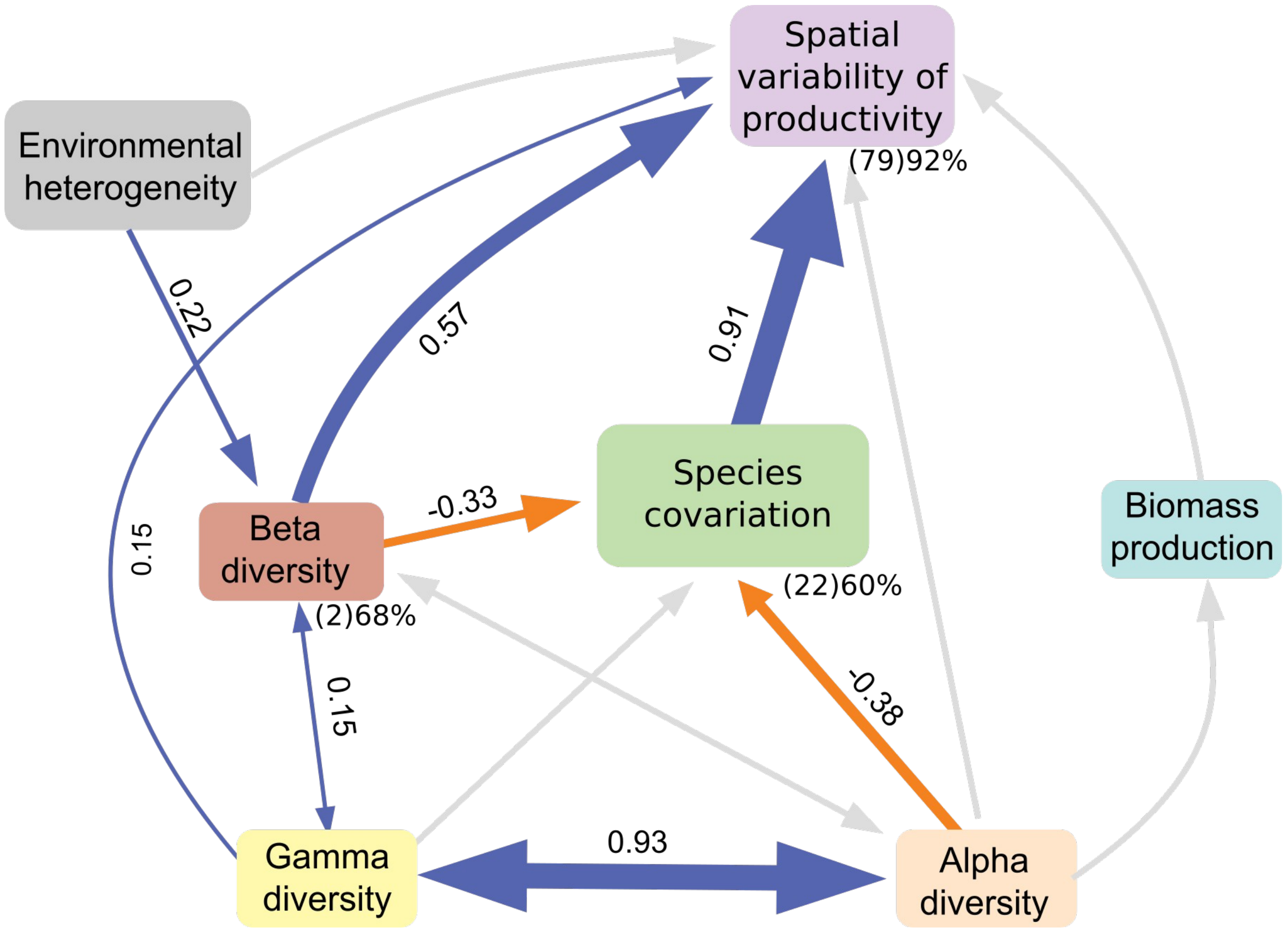


**Supplementary Fig. 4. SEM model using the subset of 54 sites in which plot level soil chemistry was measured, allowing to estimate environmental spatial heterogeneity.** Model fit was assessed using Shipley's test of d-separation (Fisher's C= 7.93, df= 8, P= 0.441). Solid blue arrows and solid orange arrows represent significant ($P \leq 0.05$, no multiple comparison adjustments made) positive and negative paths, respectively, and light gray arrows represent non-significant paths that were included in the initial model. Test of significance of path coefficients are two-sided for a difference from 0. Bidirectional arrows represent paths that were modeled as correlated errors (i.e. bidirectional relations instead of causal and unidirectional relations). Numbers next to the arrows are averaged effect sizes as standardized path coefficients (see Supplementary Table 7 for non-standardized coefficient values and exact P-values of individual paths). Width of arrows reflect these standardized effect sizes. The marginal (i.e. explained by the fixed factors alone) and conditional (i.e. explained by both the fixed and the random factors) percent variance of endogenous variables ($R^2$) are shown next to them (marginal between brackets).

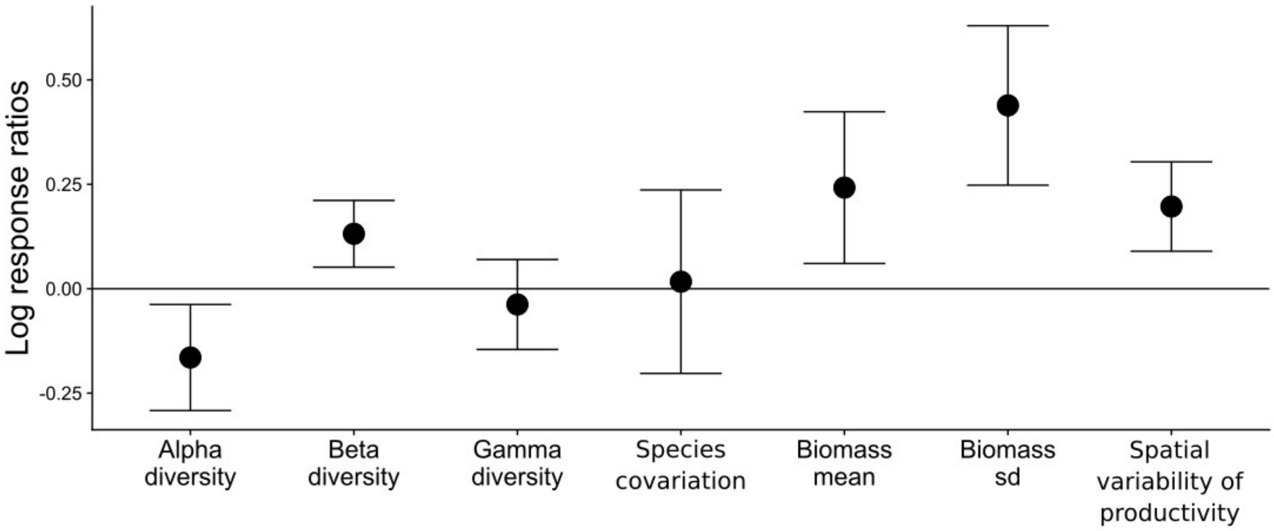


**Supplementary Fig. 5. The effect of increased heterogeneity in nutrient inputs.** Log Response Ratio (LRR) of species richness (alpha diversity), multivariate distance in species assemblages among plots within a block (beta diversity), total species richness (gamma diversity), species covariation mean and standard deviation of biomass production, and spatial variability of productivity, to 4 years of increased spatial heterogeneity in nutrient inputs at the subset of 42 sites that implemented the experimental protocol (i.e. n= 42). Increased heterogeneity drives a general increase in both the mean and the standard deviation of biomass productivity and results in a decrease in alpha diversity but an increase in beta diversity and in the spatial variability of biomass. Circles are means and error bars represent the 95% confidence interval.

**Supplementary Fig. 6**

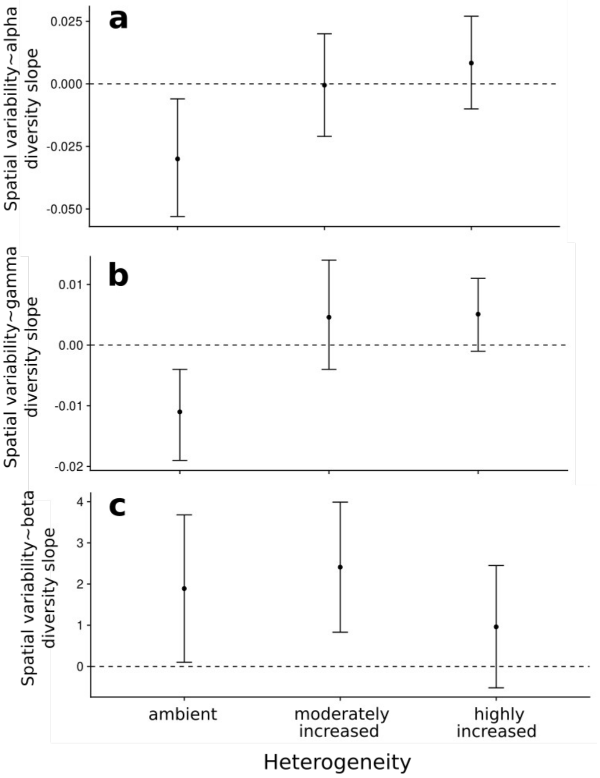


**Supplementary Fig. 6. The effect of different levels of heterogeneity on the slope of the relation between the different scales of diversity and the spatial variability of biomass production.** As our experimental design has only two imposed levels of environmental heterogeneity, we statistically shuffled plots across blocks to create scenarios consisting in artificial "blocks" with "moderately increased heterogeneity". For each of the 42 experimental sites, we grouped together 10 plots originally belonging to the 3 different blocks and collectively representing 4 different environmental conditions (e.g., the 3 control plots, the 3 +Kµ plots, the 3 +P plots and 1 +PKµ plot). Given that this rearrangement allows only one artificial block per site (composed of plots from different blocks), we had to also create one block for the remaining two levels of heterogeneity by shuffling plots of different blocks. To create the (low) "natural heterogeneity" scenarios, we randomly sampled and grouped together 10 pre-treatment plots (from the 30 plots per site that resulted by pooling together the plots of the 3 blocks). Similarly, the "highly increased heterogeneity" scenario was created by randomly choosing one of the three replicates of each of the 10 treatments, leading to an artificial block with 10 plots (one per treatment) randomly chosen from any of the 3 original blocks. For each site and level of heterogeneity, we estimated alpha, beta and gamma diversity, as well as the spatial variability of biomass as described in the main text. We then modeled the relationships of the different scales of diversity with the spatial variability of biomass with type II regression and constructed confidence intervals for the slopes of the relations using the 'lmodel2' package (see ref[66]) in R with 1000 permutations. Figure shows the estimated slope and the confidence interval (95%) for each level of heterogeneity for the relation between the spatial variability of biomass (log scale) and **a** alpha, **b** gamma and, **c** beta diversity. For illustration, the moderately increased scenario depicted in the figure correspond to the arrangement of the 3 control plots, the 3 +Kµ plots, the 3 +P plots and 1 +PKµ plot, but alternative arrangements show similar patterns. Results show that the scenarios of intermediate heterogeneity (i.e. "moderately increased heterogeneity") present intermediate slope values.

**Supplementary Fig. 7**

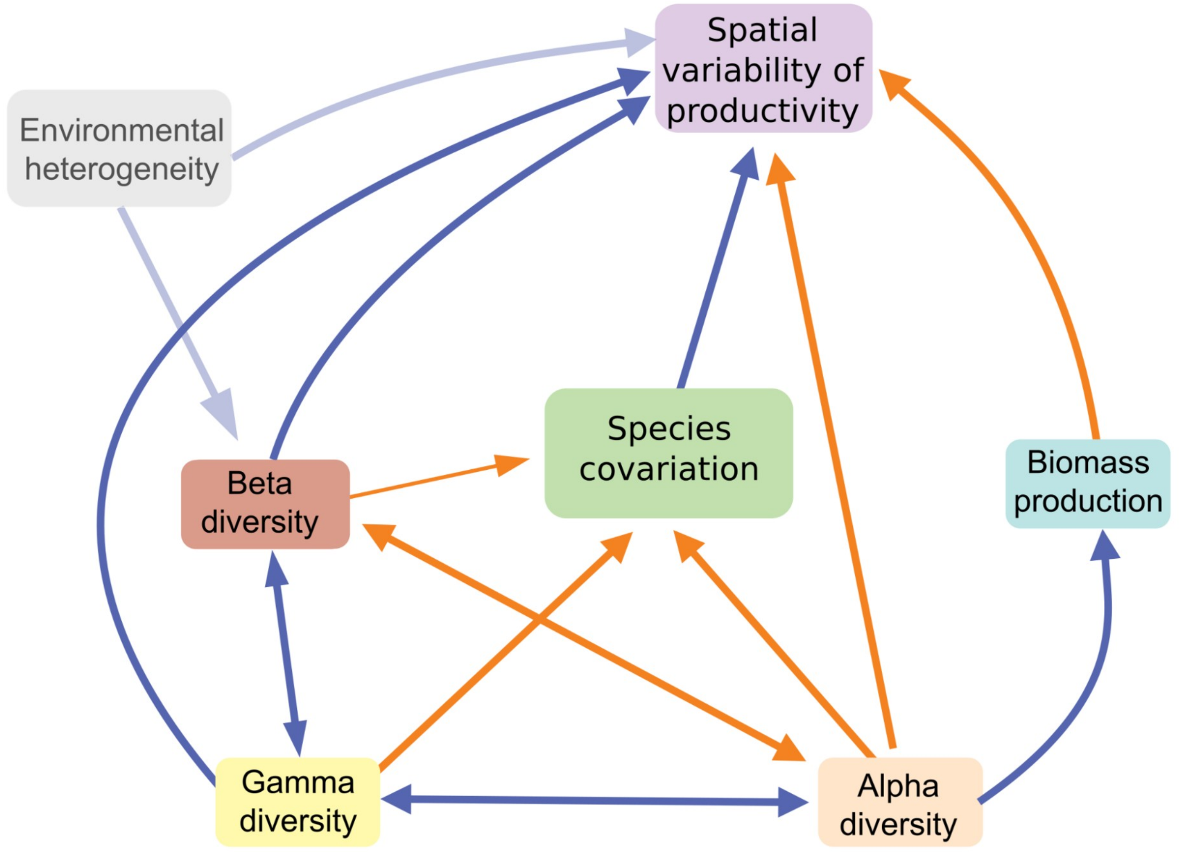


**Supplementary Fig. 7.** The *a priori* conceptual model that was used as a framework to carry out the SEM analysis to evaluate the relationship between different scales of diversity and spatial variability of biomass production (see Supplementary Table 5 for explanation of the pathways). Environmental heterogeneity and its pathways were included in a different SEM analysis using the subset of sites in which soil chemistry was measured, allowing us to start with an initial model that included spatial variability in soil conditions as a predictor of both beta diversity and spatial variability of biomass production. Blue arrows represent expected positive paths, orange arrows represent expected negative paths and bidirectional arrows represent correlated errors (i.e. bidirectional relations instead of causal and unidirectional relations).

**Supplementary Table 1. Additional information on the 83 Nutrient Network study sites.**

| Site code | Name | Continent | Country | Habitat | Post treatment | Blocks | Plots per block |
|---|---|---|---|---|---|---|---|
| ahth.is | Audkuluheidi Heath | Europe | | heathland | | 3 | 10 |
| amcamp.us | American Camp | North America | | mesic grassland | | 3 | 10 |
| amlr.is | Audkuluheidi Melur | Europe | | desert grassland | | 3 | 10 |
| anti.ec | Antisana | South America | | alpine grassland | | 5 | 10 |
| arch.us | Archbold Biological Station | North America | | mixedgrass prairie | | 3 | 10 |
| azi.cn | Azi | Asia | | alpine grassland | ✓ | 3 | 10 |
| badlau.de | Bad Lauchstaedt | Europe | | old field | ✓ | 3 | 8 |
| bari.ar | Fortín Chacabuco | South America | | grassland steppe | | 3 | 10 |
| barta.us | Barta Brothers | North America | | mixedgrass prairie | ✓ | 3 | 10 |
| bnbt.us | Benedictine Bottoms | North America | | tallgrass prairie | | 3 | 10 |
| bnch.us | Bunchgrass (Andrews LTER) | North America | | montane grassland | ✓ | 3 | 10 |
| bogong.au | Bogong | Australia | | alpine grassland | ✓ | 3 | 10 |
| bttr.us | Buttercup (Andrews LTER) | North America | | montane grassland | | 3 | 10 |
| bunya.au | Bunya Mountains | Australia | | grassland | | 3 | 10 |
| burrawan.au | Burrawan | Australia | | semiarid grassland | ✓ | 3 | 10 |
| burren.ie | Slieve Carran | Europe | | calcareous grassland | | 3 | 10 |
| bynb.cn | Bayanbulak | Asia | | alpine grassland | | 3 | 10 |
| cbgb.us | Chichaqua Bottoms | North America | | tallgrass prairie | ✓ | 6 | 10 |
| cdcr.us | Cedar Creek LTER | North America | | tallgrass prairie | ✓ | 5 | 10 |
| cdpt.us | Cedar Point Biological Station | North America | | shortgrass prairie | ✓ | 6 | 10 |
| chilcas.ar | Las Chilcas | South America | | mesic grassland | ✓ | 3 | 10 |
| cowi.ca | Cowichan | North America | | old field | ✓ | 3 | 10 |
| derr.au | Derrimut | Australia | | semiarid grassland | | 3 | 10 |
| ethamc.au | Ethabuka (Main Camp) | Australia | | desert grassland | | 3 | 10 |
| ethass.au | Ethabuka (South Site) | Australia | | desert grassland | | 3 | 10 |
| frue.ch | Fruebuel | Europe | | pasture | ✓ | 3 | 10 |
| gall.it | Galleno | Europe | | mesic grassland | | 4 | 8 |
| gilb.za | Mt Gilboa | Africa | | montane grassland | | 3 | 10 |
| glac.us | Glacial Heritage | North America | | mesic grassland | | 3 | 10 |
| glcr.us | Glacier Creek Preserve | North America | | tallgrass prairie | | 3 | 10 |
| hall.us | Hall's Prairie | North America | | tallgrass prairie | ✓ | 3 | 10 |
| hart.us | Hart Mountain | North America | | shrub steppe | ✓ | 3 | 10 |
| hast.us | Hastings UCNRS | North America | | annual grassland | | 3 | 10 |
| hnvr.us | Hanover | North America | | old field | | 3 | 10 |
| hopl.us | Hopland REC | North America | | annual grassland | ✓ | 3 | 10 |
| jasp.us | Jasper Ridge Biological Preserve | North America | | annual grassland | | 3 | 10 |
| jena.de | JeNut | Europe | | grassland | ✓ | 3 | 8 |

| Site code | Name | Continent | Country | Habitat | Post treatment | Blocks | Plots per block |
|---|---|---|---|---|---|---|---|
| kbs.us | Kellogg Biological Station LTER | North America | | old field | ✔ | 5 | 10 |
| kibber.in | Kibber (Spiti) | Asia | | alpine grassland | ✔ | 3 | 10 |
| kidman.au | Kidman Springs | Australia | | savanna | | 3 | 10 |
| kilp.fi | Kilpisjärvi | Europe | | tundra grassland | ✔ | 4 | 10 |
| kiny.au | Kinypanial | Australia | | semiarid grassland | ✔ | 3 | 10 |
| kirik.ee | Kirikukyla | Europe | | calcareous grassland | | 3 | 10 |
| koffler.ca | Koffler Scientific Reserve at Joker's Hill | North America | | pasture | ✔ | 3 | 12 |
| konz.us | Konza LTER | North America | | tallgrass prairie | ✔ | 3 | 10 |
| lake.us | Lakeside Laboratory | North America | | tallgrass prairie | ✔ | 3 | 8 |
| lancaster.uk | Lancaster | Europe | | mesic grassland | | 3 | 10 |
| lead.us | Leadbetter Point | North America | | salt marsh | | 3 | 10 |
| look.us | Lookout (Andrews LTER) | North America | | montane grassland | ✔ | 3 | 10 |
| lubb.us | Lubbock (Texas Tech Univ) | North America | | semiarid grassland | | 3 | 10 |
| marc.ar | Mar Chiquita | South America | | grassland | ✔ | 3 | 10 |
| mcdan.us | McDaniel College | North America | | NA | | 3 | 12 |
| mcla.us | Mclaughlin UCNRS | North America | | annual grassland | ✔ | 3 | 10 |
| msla.us | Missoula | North America | | grassland | | 3 | 10 |
| msum.us | Minnesota State University Moorehead | North America | | tallgrass prairie | | 3 | 10 |
| mtca.au | Mt. Caroline | Australia | | savanna | ✔ | 4 | 10 |
| nilla.au | Nillahcootie | Australia | | old field | | 3 | 10 |
| niwo.us | Niwot Ridge LTER | North America | | alpine grassland | | 4 | 9 |
| pich.ec | Pichincha | South America | | alpine grassland | | 5 | 10 |
| ping.au | Pingelly Paddock | Australia | | old field | ✔ | 3 | 10 |
| pinj.au | Pinjarra Hills | Australia | | pasture | | 3 | 10 |
| podo.ec | Podocarpus | South America | | paramo | | 3 | 10 |
| potrok.ar | Potrok Aike | South America | | semiarid grassland | | 3 | 10 |
| saana.fi | Saana | Europe | | montane grassland | ✔ | 4 | 10 |
| sage.us | Sagehen Creek UCNRS | North America | | montane grassland | ✔ | 3 | 10 |
| sedg.us | Sedgwick Reserve UCNRS | North America | | annual grassland | ✔ | 3 | 10 |
| sereng.tz | Serengeti | Africa | | savanna | ✔ | 3 | 10 |
| sevi.us | Sevilleta LTER | North America | | desert grassland | ✔ | 5 | 8 |
| shps.us | Sheep Experimental Station | North America | | shrub steppe | ✔ | 4 | 10 |
| sier.us | Sierra Foothills REC | North America | | annual grassland | ✔ | 3 | 10 |
| smith.us | Smith Prairie | North America | | mesic grassland | ✔ | 3 | 10 |
| spin.us | Spindletop | North America | | pasture | ✔ | 3 | 10 |
| spv.ar | San Pablo de Valdes | South America | | semiarid grassland | | 3 | 10 |
| summ.za | Summerveld | Africa | | mesic grassland | | 3 | 10 |

| Site code | Name | Continent | Country | Habitat | Post treatment | Blocks | Plots per block |
|---|---|---|---|---|---|---|---|
| temple.us | Temple | North America | 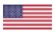 | tallgrass prairie | ✔ | 3 | 8 |
| tyso.us | Tyson | North America | 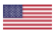 | old field | | 4 | 10 |
| ufrec.us | UF Range Cattle REC | North America | 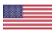 | grassland | | 3 | 8 |
| ukul.za | Ukulinga | Africa | 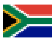 | mesic grassland | ✔ | 3 | 10 |
| unc.us | Duke Forest | North America | 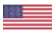 | old field | ✔ | 3 | 10 |
| uwo.ca | UWO Observatory | North America | 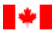 | old field | | 3 | 10 |
| valm.ch | Val Mustair | Europe | 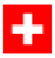 | alpine grassland | ✔ | 3 | 10 |
| veluwe.nl | Veluwe | Europe | 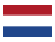 | old field | | 5 | 10 |
| yarra.au | Yarramundi | Australia | 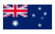 | mesic grassland | ✔ | 4 | 10 |

**Supplementary Table 2. Statistical results of linear mixed-effect models (with sites as random effect) evaluating the relation between different diversity indexes and spatial variability of ANPP in 83 NutNet grassland sites.** Inference was based on 95% confidence intervals of the slopes and on the Likelihood Ratio Test, that tests the difference in two nested models (in this case with and without the diversity index to be evaluated) using the Chi square distribution (two-sided test).

| Diversity Index | slope | 95% interval (inferior, superior) | P |
|---|---|---|---|
| Alpha | | | |
| Shannon | -0.22 | -0.35, -0.10 | 0.00115 |
| Inverse Simpson | -0.058 | -0.0090, -0.027 | 0.00044 |
| Spie | -0.03 | -0.05, -0.01 | 0.00890 |
| Gamma | | | |
| Shannon | -0.07 | -0.19, 0.05 | 0.23310 |
| Inverse Simpson | -0.02 | -0.04, -0.002 | 0.03739 |
| Beta | | | |
| Multiplicative | 0.86 | 0.58, 1.15 | $2.34*10^{-8}$ |
| Additive | -0.032 | -0.013, 0.006 | 0.47310 |
| Multivariate (abundance based) | 1.50 | 0.82, 2.19 | $1.96*10^{-5}$ |

**Supplementary Table 3. Statistical results of type II regression after removing differences in site environmental conditions.** To remove the possible influence of key abiotic factors on the relationship between different scales of diversity and spatial variability of productivity, we used a subset of bioclimatic variables representing (i) annual trends (mean annual temperature (°C) and precipitation (mm); seasonality: mean annual range in temperature (°C), standard deviation in temperature, coefficient of variation of precipitation) and (ii) extreme or limiting environmental factors (mean temperature during the wettest 4 months (°C)). We performed a multiple regression of spatial variability against these climatic variables, kept the residuals, and then modeled the relationship between different scales of diversity and the obtained residuals, using type II regression. Inference was based on 95% confidence intervals of the slopes and on the permutational probability (one-tailed, for the tail corresponding to the sign of the slope estimate).

| Diversity scale | $r^2$ | slope | 95% interval (inferior, superior) | P |
|---|---|---|---|---|
| Alpha | 0.16 | -0.049 | -0.078, -0.027 | 0.001 |
| Gamma | 0.10 | -0.021 | -0.037, -0.008 | 0.002 |
| Beta | 0.05 | 7.08 | 2.23, 24.73 | 0.018 |

**Supplementary Table 4. Statistical results of the best model (after multi-model selection) including bioclimatic variables.** To evaluate the possible influence of key abiotic factors on the relationship between different scales of diversity and spatial variability of productivity, we used a subset of bioclimatic variables representing (i) annual trends (mean annual temperature (°C) and precipitation (mm); seasonality: mean annual range in temperature (°C), standard deviation in temperature, coefficient of variation of precipitation) and (ii) extreme or limiting environmental factors (mean temperature during the wettest 4 months (°C)). We performed a multi-model inference to select the simplest models that explained the most variation (of spatial variability) based on Akaike's information criterion (AIC). Candidate models represented every possible combination of explanatory variables (i.e. the subset of bioclimatic variables along with the different scales of diversity) and the interactions between bioclimatic variables and the different scales of diversity. For each level of diversity, best model always included a single bioclimatic variable, the scale of diversity and the interaction between them. Inference was based on F-test of the selected model (two-sided test).

| Variable | df | MS | F | P |
|---|---|---|---|---|
| **Model for alpha diversity** | | | | |
| MAP | 1 | 1.20 | 7.39 | 0.008 |
| diversity | 1 | 4.41 | 27.06 | $1.5*10^{-6}$ |
| MAP*diversity | 1 | 1.04 | 6.41 | 0.01 |
| Residuals | 79 | 0.16 | | |
| **Model for beta diversity** | | | | |
| MAT_RANGE | 1 | 2.13 | 10.94 | 0.0001 |
| diversity | 1 | 0.65 | 3.80 | 0.055 |
| MAT_RANGE*diversity | 1 | 1.21 | 6.26 | 0.01 |
| Residuals | 79 | 0.19 | | |
| **Model for gamma diversity** | | | | |
| MAP | 1 | 1.20 | 6.60 | 0.01 |
| diversity | 1 | 2.72 | 14.88 | 0.0001 |
| MAP*diversity | 1 | 1.20 | 6.62 | 0.01 |
| Residuals | 79 | 0.18 | | |

**Supplementary Table 5. Hypotheses related to key predictions, from theories relating biodiversity and spatial variability, used to construct the conceptual model for SEM analysis.**

| Pathway | Mechanism and hypothesis | References |
|---|---|---|
| **RELATION BETWEEN DIVERSITY SCALES** | | |
| Gamma diversity ↔ Alpha diversity<br>Gamma diversity ↔ Beta diversity<br>Beta diversity ↔ Alpha diversity | Usually gamma diversity is positively correlated with alpha and beta diversity whereas, for a given value of gamma diversity, a negative relation between alpha and beta diversity is expected. | 1 |
| **OVERYIELDING OR ENHANCED PERFORMANCE HYPOTHESIS PATHWAYS** | | |
| Alpha diversity → Biomass production | Diversity can increase productivity as a result of resource partitioning, facilitation or sampling effect. | 2, 3 |
| Biomass production → Spatial variability of productivity | When mixture biomass production exceeds the expected value based on monocultures, high diversity can increase the mean of biomass production, relative to its variance. | 4,5 |
| **INSURANCE HYPOTHESIS PATHWAYS** | | |
| Alpha diversity → Species covariation<br>Gamma diversity → Species covariation | We estimated species covariation across space as a spatial analogous of species synchrony. Species that are redundant or functionally insignificant under certain environmental conditions, can be no longer redundant or insignificant if environmental conditions change. Thus, if different species are capable to differently respond to environmental variability, high species diversity decreases species covariation. | 6-10 |
| Beta diversity → Species covariation | If different species are capable to differently respond to environmental variability, higher variation and dissimilarity in species composition among communities may decrease species covariation. | 11 |
| Species covariation → Spatial variability of productivity | Declines in the abundance of some species are compensated for by increases in others, thus buffering | 12, 13 |

| | | |
|---|---|---|
| | spatial fluctuation in the productivity of the whole community. | |
| **OTHER PATHWAYS** | | |
| Alpha diversity → Spatial variability of productivity<br><br>Gamma diversity → Spatial variability of productivity | The effect of diversity on the spatial variability of productivity could be related to other mechanisms, such as observation error (the random effects of uncontrolled factors) among others. | 14 |
| Beta diversity → Spatial variability of productivity | Beta diversity can increase spatial variability of productivity as it can imply shifts in functional traits that scale up to affect community production | 15-17 |
| Spatial environmental heterogeneity → beta diversity | Spatial heterogeneity in environmental conditions may generate divergence in species composition among local communities or patches (i.e. high beta diversity caused by species sorting) that may indirectly increase gamma diversity) | 18, 19 |
| Spatial environmental heterogeneity → Spatial variability of biomass production | Plants are affected by environmental factors during all phases of growth and development. A major cause of spatial variability in productivity is small-scale heterogeneity of abiotic site conditions, thus, spatial environmental heterogeneity may imply differences in plant productivity. | 20 |

**Supplementary Table 6. Non-standardized coefficients and probability of individual paths of the SEM analysis to infer the direct and indirect effects of biodiversity on the spatial variability of productivity.** Environmental heterogeneity → beta diversity path was estimated using the subset of 54 sites in which plot level soil chemistry was measured, allowing to estimate environmental spatial heterogeneity (see Supplementary Figure 4).

| Path | Coefficient | P |
|---|---|---|
| Gamma diversity → Spatial variability of productivity | 0.0044 | 0.0285 |
| Beta diversity → Spatial variability of productivity | 1.8823 | $1.38*10^{-9}$ |
| Species covariation → Spatial variability of productivity | 0.4904 | $<0.01*10^{-100}$ |
| Biomass production → Spatial variability of productivity | -0.0963 | 0.0002 |
| Alpha diversity → Species covariation | -0.0644 | $2.47*10^{-10}$ |
| Beta diversity → Species covariation | -1.7854 | 0.0192 |
| Gamma diversity ↔ Alpha diversity | 0.9192 | $<0.01*10^{-100}$ |
| Gamma diversity ↔ Beta diversity | 0.2110 | 0.0004 |
| Environmental heterogeneity → Beta diversity | 0.0333 | 0.0424 |

**Supplementary Table 7. Exact P-values of the multigroup analysis.** This analysis implements a model-wide interaction in which every term in the model interacts with the grouping variable (i.e. pre- versus post-treatment). If the interaction is significant, then the path is free to vary by group; if not, then the path is constrained and takes on the estimate from the global dataset.

| Path | P |
|---|---|
| Gamma diversity → Spatial variability of productivity | 0.0628 |
| Beta diversity → Spatial variability of productivity | 0.9253 |
| Species covariation → Spatial variability of productivity | 0.7180 |
| Alpha diversity → Species covariation | 0.0001 |
| Beta diversity → Species covariation | 0.0116 |

**Supplementary Table 8. Non-standardized coefficients and probability of individual paths of the comparison of SEM models with ambient and experimentally increased spatial heterogeneity, using the subset of 42 sites that implemented the experimental protocol.** P-values followed for a (c) denotes path coefficients that have been constrained (multigroup analysis; P >0.05); path coefficients are globally estimated and P-values are thus the same for Ambient and Experimentally increased heterogeneity, but standardized coefficients differ because the variance differs between groups, and thus the standardization. P-values followed for a (*) denotes path coefficients that differ between pre- and post-treatment models (multigroup analysis; P <0.05; see Supplementary Table 7 for exact P-values).

| Path | Coefficient | P |
|---|---|---|
| **Ambient heterogeneity** | | |
| Gamma diversity → Spatial variability of productivity | 0.0057 | 0.0014 (c) |
| Beta diversity → Spatial variability of productivity | 2.0132 | $2.89*10^{-12}$ (c) |
| Species covariation → Spatial variability of productivity | 0.4592 | $<0.01*10^{-100}$ (c) |
| Alpha diversity → Species covariation | -0.0621 | $1.49*10^{-5}$ * |
| Beta diversity → Species covariation | -0.6007 | 0.5894 * |
| Gamma diversity ↔ Alpha diversity | 0.8835 | $5.97*10^{-48}$ |
| Gamma diversity ↔ Beta diversity | 0.2478 | 0.0012 |
| **Experimentally increased heterogeneity** | | |
| Gamma diversity → Spatial variability of productivity | 0.0057 | 0.0014 (c) |
| Beta diversity → Spatial variability of productivity | 2.0132 | $2.89*10^{-12}$ (c) |
| Species covariation → Spatial variability of productivity | 0.4592 | $<0.01*10^{-100}$ (c) |
| Alpha diversity → Species covariation | -0.0153 | 0.2131 * |
| Beta diversity → Species covariation | -4.0219 | $3.81*10^{-5}$ * |
| Gamma diversity ↔ Alpha diversity | 0.9089 | $2.07*10^{-58}$ |
| Gamma diversity ↔ Beta diversity | 0.2295 | 0.0060 |

**Supplementary References**


1. Whittaker, R. H. Vegetation of the Siskiyou mountains, Oregon and California. *Ecol. Monogr.* **30**, 279– 338 (1960).

2. Tilman, D. *et al.* Plant diversity and ecosystem productivity: theoretical considerations. *Proc. Natl. Acad. Sci.* **94**, 1857–1861 (1997).

3. Loreau, M. & Hector, A. Partitioning selection and complementarity in biodiversity experiments. *Nature* **412**, 72–76 (2001).

4. Lehman, C. L. & Tilman, D. Biodiversity, Stability, and Productivity in Competitive Communities. *Am. Nat.* **156**, 534–552 (2000).

5. Yachi, S. & Loreau, M. Biodiversity and ecosystem productivity in a fluctuating environment: The insurance hypothesis. *Proc. Natl. Acad. Sci.* **96**, 1463–1468 (1999).

6. McCann, K. S. The diversity–stability debate. *Nature* **405**, 228–233 (2000).

7. Loreau, M. *et al.* Biodiversity and ecosystem functioning: current knowledge and future challenges. *Science* **294**, 804–808 (2001).

8. Isbell, F. *et al.* Biodiversity increases the resistance of ecosystem productivity to climate extremes. *Nature* 526, 574–577 (2015).

9- Wang, S. & Loreau, M. Ecosystem stability in space: α, β and γ variability. *Ecol. Lett.* **17**, 891–901 (2014).

10. Hautier, Y. *et al.* Eutrophication weakens stabilizing effects of diversity in natural grasslands. *Nature* **508**, 521–525 (2014).

11. Allan, E. *et al.* More diverse plant communities have higher functioning over time due to turnover in complementary dominant species. *Proc. Natl. Acad. Sci.* **108**, 17034–17039 (2011).

12. Ives, A. R., Gross, K. & Klug, J. L. Stability and Variability in Competitive Communities. *Science* **286**, 542–544 (1999).

13. Loreau, M., Mouquet, N. & Gonzalez, A. Biodiversity as spatial insurance in heterogeneous landscapes. *Proc. Natl. Acad. Sci.* **100**, 12765–12770 (2003).

14. de Mazancourt, C. *et al.* Predicting ecosystem stability from community composition and biodiversity. *Ecol. Lett.* **16**, 617-625 (2013).

15. La Pierre, K. J. & Smith, M. D. Functional trait expression of grassland species shift with short- and long-term nutrient additions. *Plant Ecol.* **216**, 307–318 (2015).

16. Koerner, S. E. *et al.* Nutrient additions cause divergence of tallgrass prairie plant communities resulting in loss of ecosystem stability. *J. Ecol.* **104**, 1478–1487 (2016).

17. Wang, S. & Loreau, M. Biodiversity and ecosystem stability across scales in metacommunities. *Ecol. Lett.* **19**, 510–518 (2016).

18. Tamme, R. *et al.* Environmental heterogeneity, species diversity and co-existence at different spatial scales. *J. Veg. Sci.* **21**, 796–801 (2010).

19. Hodapp, D. *et al.* Spatial heterogeneity in species composition constrains plant community responses to herbivory and fertilisation. *Ecol. Lett.* **21**, 1364–1371 (2018).

20. Weigelt, A. *et al.* Does biodiversity increase spatial stability in plant community biomass? *Ecol. Lett.* **11**, 338–347 (2008).
