# Supplementary material for: Environmental heterogeneity modulates the effect of plant diversity on the spatial variability of grassland biomass
Source: Nat Commun. 2023 Mar 31;14:1809. doi: 10.1038/s41467-023-37395-y (PMC10066197; doi:10.1038/s41467-023-37395-y)
Supplement: Supplementary file 3 — Reporting Summary [file 41467_2023_37395_MOESM3_ESM.pdf]

nature portfolio

Corresponding author(s): Pedro Daleo

Last updated by author(s): Mar 8, 2023

Reporting Summary

Nature Portfolio wishes to improve the reproducibility of the work that we publish. This form provides structure for consistency and transparency in reporting. For further information on Nature Portfolio policies, see our Editorial Policies and the Editorial Policy Checklist.

Statistics

For all statistical analyses, confirm that the following items are present in the figure legend, table legend, main text, or Methods section.

n/a	Confirmed	
☐	☒	The exact sample size (*n*) for each experimental group/condition, given as a discrete number and unit of measurement
☐	☒	A statement on whether measurements were taken from distinct samples or whether the same sample was measured repeatedly
☐	☒	The statistical test(s) used AND whether they are one- or two-sided<br>*Only common tests should be described solely by name; describe more complex techniques in the Methods section.*
☐	☒	A description of all covariates tested
☐	☒	A description of any assumptions or corrections, such as tests of normality and adjustment for multiple comparisons
☐	☒	A full description of the statistical parameters including central tendency (e.g. means) or other basic estimates (e.g. regression coefficient) AND variation (e.g. standard deviation) or associated estimates of uncertainty (e.g. confidence intervals)
☐	☒	For null hypothesis testing, the test statistic (e.g. *F*, *t*, *r*) with confidence intervals, effect sizes, degrees of freedom and *P* value noted<br>*Give P values as exact values whenever suitable.*
☒	☐	For Bayesian analysis, information on the choice of priors and Markov chain Monte Carlo settings
☐	☒	For hierarchical and complex designs, identification of the appropriate level for tests and full reporting of outcomes
☐	☒	Estimates of effect sizes (e.g. Cohen's *d*, Pearson's *r*), indicating how they were calculated

Our web collection on statistics for biologists contains articles on many of the points above.

Software and code

Policy information about availability of computer code

Data collection	Data comes from the Nutrient Network, a coordinated, multi-site and multi-year nutrient enrichment experiment (nitrogen, phosphorous and potassium addition). Data were retrieved on may 6th 2021. All data used in these analyses are publicly available on the Environmental Data Initiative (EDI) (doi:10.6073/pasta/583874460a0af70f93d3eee2f22f9a13). In addition, the complete R code supporting the findings of this study is freely available online at GitHub and archived through Zenodo (doi: 10.5281/zenodo.7698668) No software was used for data colection.
Data analysis	All analyses were conducted in R 4.0.5

For manuscripts utilizing custom algorithms or software that are central to the research but not yet described in published literature, software must be made available to editors and reviewers. We strongly encourage code deposition in a community repository (e.g. GitHub). See the Nature Portfolio guidelines for submitting code & software for further information.

Data

Policy information about availability of data

All manuscripts must include a data availability statement. This statement should provide the following information, where applicable:

- Accession codes, unique identifiers, or web links for publicly available datasets
- A description of any restrictions on data availability
- For clinical datasets or third party data, please ensure that the statement adheres to our policy

All data used in these analyses are publicly available on the Environmental Data Initiative (EDI) (doi:10.6073/pasta/583874460a0af70f93d3eee2f22f9a13)

Human research participants

Policy information about studies involving human research participants and Sex and Gender in Research.

Reporting on sex and gender	*Use the terms sex (biological attribute) and gender (shaped by social and cultural circumstances) carefully in order to avoid confusing both terms. Indicate if findings apply to only one sex or gender; describe whether sex and gender were considered in study design whether sex and/or gender was determined based on self-reporting or assigned and methods used. Provide in the source data disaggregated sex and gender data where this information has been collected, and consent has been obtained for sharing of individual-level data; provide overall numbers in this Reporting Summary. Please state if this information has not been collected. Report sex- and gender-based analyses where performed, justify reasons for lack of sex- and gender-based analysis.*
Population characteristics	*Describe the covariate-relevant population characteristics of the human research participants (e.g. age, genotypic information, past and current diagnosis and treatment categories). If you filled out the behavioural & social sciences study design questions and have nothing to add here, write "See above."*
Recruitment	*Describe how participants were recruited. Outline any potential self-selection bias or other biases that may be present and how these are likely to impact results.*
Ethics oversight	*Identify the organization(s) that approved the study protocol.*

Note that full information on the approval of the study protocol must also be provided in the manuscript.

Field-specific reporting

Please select the one below that is the best fit for your research. If you are not sure, read the appropriate sections before making your selection.

☐ Life sciences ☐ Behavioural & social sciences ☒ Ecological, evolutionary & environmental sciences

For a reference copy of the document with all sections, see nature.com/documents/nr-reporting-summary-flat.pdf

Ecological, evolutionary & environmental sciences study design

All studies must disclose on these points even when the disclosure is negative.

Study description	We used observational data from 83 grassland sites that are part of the Nutrient Network (NutNet) Global Research Cooperative, covering a wide range of grassland habitats and relevant gradient of fine-scale and site-level variation. A subset of 42 of those sites implemented, for at least 4 years, an experiment with three nutrient addition treatments (N, P and K). Treatments were randomly assigned to the plots and were replicated in three blocks at most sites (some sites used in this study had more blocks). See http://www.nutnet.org for more details.
Research sample	Plant productivity, cover, and diversity from 83 grassland sites across the globe (https://nutnet.org/).
Sampling strategy	Sampling was done in 1 m2 subplots and followed a standardized protocol at all sites. The number of samples was chosen to allow for analyses of all data, but to minimize the efforts for sampling at each site.
Data collection	Treatments and sampling followed a standardized protocol at all sites, detailed in (Borer, E. T. et al. Finding generality in ecology: a model for globally distributed experiments. Methods in Ecology and Evolution 5, 63-73 (2013)). Primary productivity was estimated annually by clipping at ground level using grass shears all aboveground live biomass from two 0.1 m2 (10 x 100 cm) quadrats per subplot. Areal percent cover of each species was measured concurrently with primary productivity in one 1 x 1m subplot in which no destructive sampling occurred. Cover and primary productivity were estimated twice during the year at some sites with strongly seasonal communities. We quantified local scale and larger scale diversity indices across the three replicated 1-m2 subplots for each site, treatment and duration period using cover data. Data collection was done by the principal investigator at each site.

Timing and spatial scale

Sampling of observational data was done between 2008 and 2020. Treatment application started at most sites in 2008, but some sites started later. Plots at all sites were 5 × 5 m (separated by at least 1 m walkways) spread over an area of at least 1000 m2. Sampling was done in 1 m2 plots grouped into spatial blocks spread over 320 m2.

Data exclusions

From the Nutrient Network, we selected sites that had at least three blocks and at least 8 plots per block. For the experimental data we selected sites that implemented, for at least 4 years, an experiment with three nutrient addition treatments. Exclusion criteria were pre-established.

Reproducibility

We have used standard experimantal and sampling methods in community ecology and have carefully reported how the sampling and experimental manipulation was done. Data were retrieved on 6 May 2021 from the NutNet dropbox (updated regularly by the data manager).

Randomization

Treatments were randomly assigned.

Blinding

The principal investigator at each site knows the location of each treatment (because it is necesary). However, data were analyzed using all replicates per site, but without knowing which site had which influence on the overall results.

Did the study involve field work? ☒ Yes ☐ No

Field work, collection and transport

Field conditions

Ours sites were distributed over 6 continents and 18 countries. All sites are dominated by herbaceous species, and together cover a wide range of grassland habitats that range from alpine grassland, to prairie, pasture, shrub steppe, savanna and old field. These grasslands also cover a wide range in elevation (0 to 4400 masl), mean annual precipitation (192 to 2566 mm yr-1), mean annual temperature (-7 to 27° C), latitude (52 degrees S to 69 degrees N), and aboveground productivity (0.5 to 1445 g m-2 yr-1). See Fig. 2B.

Location

Site code, site name, continent, latitude, longitude and grassland type at each site can be found below (as well as in Fig. 2B and Supplementary Table S1).

ahth.is, Audkuluheidi Heath, Europe, 65.1, -19.7, heathland
amcamp.us, American Camp, North America, 48.5, -123.01, mesic grassland
amlr.is, Audkuluheidi Melur, Europe, 65.1, -19.7, desert grassland
anti.ec, Antisana, South America, -0.5, -78.2, alpine grassland
arch.us, Archbold Biological Station, North America, 27.1, -81.2, mixedgrass prairie
azi.cn, Azi, Asia, 33.7, 101.9, alpine grassland
badlau.de, Bad Lauchstaedt, Europe, 51.4, 11.9, old field
bari.ar, Fortín Chacabuco, South America, -41.0, -71.2, grassland steppe
barta.us, Barta Brothers, North America, 42.2, -99.7, mixedgrass prairie
bnbt.us, Benedictine Bottoms, North America, 39.6, -95.1, tallgrass prairie
bnch.us, Bunchgrass (Andrews LTER), North America, 44.3, -121.9, montane grassland
bogong.au, Bogong, Australia, -36.9, 147.3, alpine grassland
bttr.us, Buttercup (Andrews LTER), North America, 44.3, -121.9, montane grassland
bunya.au, Bunya Mountains, Australia, -26.9, 151.6, grassland
burrawan.au, Burrawan, Australia, -27.7, 151.1, semiarid grassland
burren.ie, Slieve Carran, Europe, 53.1, -9.0, calcareous grassland
bynb.cn, Bayanbulak, Asia, 42.9, 82.7, alpine grassland
cbgb.us, Chichaqua Bottoms, North America, 41.8, -93.4, tallgrass prairie
cdcr.us, Cedar Creek LTER, North America, 45.4, -93.2, tallgrass prairie
cdpt.us, Cedar Point Biological Station, North America, 41.2, -101.6, shortgrass prairie
chilcas.ar, Las Chilcas, South America, -36.3, -58.3, mesic grassland
cowi.ca, Cowichan, North America, 48.8, -123.6, old field
derr.au, Derrimut, Australia, -37.8, 144.8, semiarid grassland
ethamc.au, Ethabuka (Main Camp), Australia, -23.8, 138.5, desert grassland
ethass.au, Ethabuka (South Site), Australia, -23.7, 138.4, desert grassland
frue.ch, Fruebuel, Europe, 47.1, 8.6, pasture
gall.it, Galleno, Europe, 43.8, 10.7, mesic grassland
gilb.za, Mt Gilboa, Africa, -29.3, 30.3, montane grassland
glac.us, Glacial Heritage, North America, 46.9, -123.0, mesic grassland
glcr.us, Glacier Creek Preserve, North America, 41.3, -96.1, tallgrass prairie
hall.us, Hall's Prairie, North America, 36.9, -86.7, tallgrass prairie
hart.us, Hart Mountain, North America, 42.7, -119.5, shrub steppe
hast.us, Hastings UCNRS, North America, 36.2, -121.5, annual grassland
hnvr.us, Hanover, North America, 43.4, -72.1, old field
hopl.us, Hopland REC, North America, 39.0, -123.1, annual grassland
jasp.us, Jasper Ridge Biological Preserve, North America, 37.4, -122.2, annual grassland

jena.de, JeNut, Europe, 50.9, 11.5, grassland
kbs.us, Kellogg Biological Station LTER, North America, 42.4, -85.4, old field
kibber.in, Kibber (Spiti), Asia, 32.3, 78.0, alpine grassland
kidman.au, Kidman Springs, Australia, -16.1, 130.9, savanna
kilp.fi, Kilpisjärvi, Europe, 69.1, 20.9, tundra grassland
kiny.au, Kinypanial, Australia, -36.2, 143.7, semiarid grassland
kirik.ee, Kirikukyla, Europe, 58.7, 23.8, calcareous grassland
koffler.ca, Koffler Scientific Reserve at Joker's Hill, North America, 44.0, -79.5, pasture
konz.us, Konza LTER, North America, 39.1, -96.6, tallgrass prairie
lake.us, Lakeside Laboratory, North America, 43.4, -95.2, tallgrass prairie
lancaster.uk, Lancaster, Europe, 54.0, -2.6, mesic grassland
lead.us, Leadbetter Point, North America, 46.6, -124.0, salt marsh
look.us, Lookout (Andrews LTER), North America, 44.2, -122.1, montane grassland
lubb.us, Lubbock (Texas Tech Univ), North America, 33.6, -101.9, semiarid grassland
marc.ar, Mar Chiquita, South America, -37.7, -57.4, grassland
mcdan.us, McDaniel College, North America, 39.5, -77.0, NA
mcla.us, Mclaughlin UCNRS, North America, 38.9, -122.4, annual grassland
msla.us, Missoula, North America, 46.7, -114.0, grassland
msum.us, Minnesota State University Moorehead, North America, 46.9, -96.5, tallgrass prairie
mtca.au, Mt. Caroline, Australia, -31.8, 117.6, savanna
nilla.au, Nillahcootie, Australia, -36.9, 146.0, old field
niwo.us, Niwot Ridge LTER, North America, 40.0, -105.4, alpine grassland
pich.ec, Pichincha, South America, -0.1, -79.0, alpine grassland
ping.au, Pingelly Paddock, Australia, -32.5, 117.0, old field
pinj.au, Pinjarra Hills, Australia, -27.5, 152.9, pasture
podo.ec, Podocarpus, South America, -4.1, -79.2, paramo
potrok.ar, Potrok Aike, South America, -51.9, -70.4, semiarid grassland
saana.fi, Saana, Europe, 69.0, 20.8, montane grassland
sage.us, Sagehen Creek UCNRS, North America, 39.4, -120.2, montane grassland
sedg.us, Sedgwick Reserve UCNRS, North America, 34.7, -120.0, annual grassland
sereng.tz, Serengeti, Africa, -2.3, 34.5, savanna
sevi.us, Sevilleta LTER, North America, 34.4, -106.7, desert grassland
shps.us, Sheep Experimental Station, North America, 44.2, -112.2, shrub steppe
sier.us, Sierra Foothills REC, North America, 39.2, -121.3, annual grassland
smith.us, Smith Prairie, North America, 48.2, -122.6, mesic grassland
spin.us, Spindletop, North America, 38.1, -84.5, pasture
spv.ar, San Pablo de Valdes, South America, -42.7, -64.2, semiarid grassland
summ.za, Summerveld, Africa, -29.8, 30.7, mesic grassland
temple.us, Temple, North America, 31.0, -97.3, tallgrass prairie
tyso.us, Tyson, North America, 38.5, -90.6, old field
ufrec.us, UF Range Cattle REC, North America, 27.4, -81.9, grassland
ukul.za, Ukulinga, Africa, -29.7, 30.4, mesic grassland
unc.us, Duke Forest, North America, 36.0, -79.0, old field
uwo.ca, UWO Observatory, North America, 43.2, -81.3, old field
valm.ch, Val Mustair, Europe, 46.6, 10.4, alpine grassland
veluwe.nl, Veluwe, Europe, 52.1, 5.8, old field
yarra.au, Yarramundi, Australia, -33.6, 150,7, mesic grassland

Access & import/export	Access to sites and data collection followed standard practices and complies with laws. No permits were requiered.
Disturbance	At each site walkways were established to minimize trampling effects on the sampling plots.

Reporting for specific materials, systems and methods

We require information from authors about some types of materials, experimental systems and methods used in many studies. Here, indicate whether each material, system or method listed is relevant to your study. If you are not sure if a list item applies to your research, read the appropriate section before selecting a response.

Materials & experimental systems

n/a	Involved in the study
☒	☐ Antibodies
☒	☐ Eukaryotic cell lines
☒	☐ Palaeontology and archaeology
☒	☐ Animals and other organisms
☒	☐ Clinical data
☒	☐ Dual use research of concern

Methods

n/a	Involved in the study
☒	☐ ChIP-seq
☒	☐ Flow cytometry
☒	☐ MRI-based neuroimaging
